# Kinect V2-Based Gait Analysis for Children with Cerebral Palsy: Validity and Reliability of Spatial Margin of Stability and Spatiotemporal Variables

**DOI:** 10.3390/s21062104

**Published:** 2021-03-17

**Authors:** Yunru Ma, Kumar Mithraratne, Nichola Wilson, Yanxin Zhang, Xiangbin Wang

**Affiliations:** 1 Department of Exercise Sciences, The University of Auckland, Auckland 1142, New Zealand; y.ma@auckland.ac.nz; 2 Auckland Bioengineering Institute, The University of Auckland, Auckland 1142, New Zealand; p.mithraratne@auckland.ac.nz; 3 Department of Surgery, The University of Auckland, Auckland 1142, New Zealand; n.wilson@auckland.ac.nz; 4 College of Rehabilitation Medicine, Fujian University of Traditional Chinese Medicine, Fuzhou 350122, China; 5 Key Laboratory of Orthopedics and Traumatology of Traditional Chinese Medicine and Rehabilitation, Fujian University of Traditional Chinese Medicine, Ministry of Education, Fuzhou 350122, China

**Keywords:** cerebral palsy, spatiotemporal, margin of stability, reliability, validity, Kinect V2

## Abstract

Children with cerebral palsy (CP) have high risks of falling. It is necessary to evaluate gait stability for children with CP. In comparison to traditional motion capture techniques, the Kinect has the potential to be utilised as a cost-effective gait stability assessment tool, ensuring frequent and uninterrupted gait monitoring. To evaluate the validity and reliability of this measurement, in this study, ten children with CP performed two testing sessions, of which gait data were recorded by a Kinect V2 sensor and a referential Motion Analysis system. The margin of stability (MOS) and gait spatiotemporal metrics were examined. For the spatiotemporal parameters, intraclass correlation coefficient (ICC_2,k_) values were from 0.83 to 0.99 between two devices and from 0.78 to 0.88 between two testing sessions. For the MOS outcomes, ICC_2,k_ values ranged from 0.42 to 0.99 between two devices and 0.28 to 0.69 between two test sessions. The Kinect V2 was able to provide valid and reliable spatiotemporal gait parameters, and it could also offer accurate outcome measures for the minimum MOS. The reliability of the Kinect V2 when assessing time-specific MOS variables was limited. The Kinect V2 shows the potential to be used as a cost-effective tool for CP gait stability assessment.

## 1. Introduction

Postural control is the ability to control the body’s position in space to achieve stability, requiring the centre of mass (COM) to be positioned within the boundary of the base of support (BOS) [1]. In the case of cerebral palsy (CP), deteriorations and delays in motor skill acquisition and development are associated with weak postural control schema, which is a significant component of gait disorders [2,3]. Children with CP are prone to fall. The fall rate of inpatient children with CP was reported to be 27% in a prospective study [4]. Approximately 35% of children reported daily falling, and 30% of them fell monthly or weekly according to a retrospective study [5]. Although most falls cause few consequences with no more than cuts and bruises, these nonfatal injuries could lead to a significant healthcare burden with increased pain, disability, length of stay, absence for treatment, and financial expenses [6].

About 50% of the falls happen during walking [7]. Children with CP tend to walk inefficiently with a larger gait velocity and stride length and demonstrate an inappropriate coronal foot placement [8]. Besides, they generate more cadences, trunk compensations, and conservative lateral postural control strategies to reestablish stability when encountering perturbances during gait [9,10,11]. Improved walking ability, such as less proneness to falls, positively affects the accomplishment of life habits and social integration [12,13]. Therefore, optimising walking ability and mobility is one of the therapeutic goals for patients with CP. Various rehabilitation programmes have been implemented to achieve this goal. For example, substantial evidence has proven that functional gait training that involves diverse interventions has clinically significant benefits on children and young adults with CP [14]. To obtain in-depth knowledge about the effect of gait rehabilitation programmes on patients with CP, quantitatively measuring gait characteristics is needed [15]. Besides, quantitative aspects of gait can directly reflect the impaired postural control ability that influences gait stability [16]. The relationship between gait quantitative features and fall risks can facilitate gait monitoring and help researchers develop targeted intervention programmes [17]. Some spatiotemporal gait outcomes, such as gait speed, walking endurance, and step length, are widely utilised among studies to assess gait stability and evaluate treatment outcomes [14,15,18,19,20].

The margin of stability (MOS) is also one of the quantitative gait measurements that evaluate the COM’s motion relative to the BOS that determined by the foot placement [21]. The MOS calculation is based on the inverted pendulum model representing the human body’s upright posture [22]. According to Hof et al.’s method, MOS is calculated as the distance between the edge of BOS and the vertical projection of the extrapolated COM, which is defined as the velocity of COM relative to the height of COM over BOS [21]. An individual is considered unstable or with a high risk of falling when the extrapolated COM exceeds BOS boundaries (e.g., MOS is negative). From the biomechanical perspective, MOS represents the “initial condition” when the subject encounters a perturbation. Compared with other stability assessment approaches that only quantify average measures, MOS is able to evaluate dynamic stability at a specific time step during locomotion [9,23].

With this advantage, MOS has already been widely applied to evaluate gait abnormality and fall risks in a wide range of clinical populations, including patients with stroke [24], Parkinson’s Disease [25], hereditary spastic paraparesis [26], as well as CP [9,27]. In a recent prospective observational study, Mehdizadeh et al. found that minimum mediolateral MOS was the most important hallmark when assessing the short-term fall risk for patients with dementia [16]. A retrospective study demonstrated that MOS was significantly correlated with the fall history of patients with multiple sclerosis, and it could be considered as an effective measure to assess dynamic stability during gait [28].

Generally, parameters required for MOS computation are recorded by optoelectronic motion capture systems combined with force plates. However, these high-end infrared cameras are costly and unfriendly to nonspecialists. Its marker-based tracking strategy may cause unnatural performance for children with CP and make marker placement a challenging job. Therefore, there is a growing need to develop cost-effective and portable gait analysis systems for clinic-based gait progression monitoring and home-based gait investigation, which can empower patients to take an active role in their health management, provide convenient and accessible options for people living in remote areas [29], and also ensure uninterruptable longitudinal self-monitoring during the epidemic time.

The Microsoft Kinect sensor, which was initially designed as a motion-sensing input device for interactive games, can perform real-time motion capture without any passive markers or handheld controllers. Compared to other innovative techniques, such as activity-tracking devices or wearable accelerometer-based devices, the Kinect has the advantage of providing unobtrusive investigation [30]. Previous studies have proposed Kinect-based gait stability assessment systems to monitor fall risks for geriatric populations [16,31]. However, very little is known about the Kinect’s validity for estimating MOS for a pediatric group like CP. Considering the relative accurate spatiotemporal tracking accuracy [32,33], it seems that MOS variables could be accurately estimated due to their spatiotemporal-related features. Since clinical measures should be valid, reliable, and responsive to meaningful changes when used with relation to interventions [13], it is logical and necessary to determine the validity and reliability of MOS derived from the Kinect before it could be used in real clinical scenarios. From the clinical aspect, the same outcome measure’s validity and reliability could differ among different patient groups [13,34]. Although the accuracy of gait spatiotemporal parameters estimated by the Kinect has been examined among adults with or without abnormal gait patterns [32,35,36], evidence involving the CP population is very limited [37]. Moreover, many more studies only covered the Kinect’s validity rather than both validity and reliability, which may hamper its application in real practice.

To fill the gaps mentioned above, we proposed a method to compute MOS parameters from skeleton landmarks tracked by the Kinect V2 sensor. The results were compared against data measured by a referential optoelectronic Motion Analysis system. This study aimed to assess the concurrent validity and interday reliability of the Kinect V2 when assessing MOS and spatiotemporal parameters of overground gait for children with CP. We hypothesised that the Kinect V2 could provide accurate and reliable measurements for MOS and spatiotemporal gait parameters.

The rest of the paper is as follows: “Methods” section details the characteristics of ten CP participants, the definition of selected gait spatiotemporal parameters, the method of MOS calculation, as well as statistical methods implemented to determine the validity and reliability of the Kinect V2 sensor; “Results” section describes the gait spatiotemporal and MOS parameters estimated by per device on per testing day. The between-device and between-testing day comparison is given in this section. “Discussion” section discusses the findings that arose from the results, analyses possible reasons that lead to the between-device and between-testing day deviations, and discuss the Kinect’s potential implementation in gait stability management for patients with CP, followed by the conclusive remarks in the “Conclusion” section.

## 2. Methods

### 2.1. Participants

Ten children with CP (three males and seven females, age = 6.4 ± 2.2 years, body mass = 23.2 ± 7.4 kg, body height = 116.7 ± 11.0 cm) were recruited and presented for the gait data collection. Participants were diagnosed as hemiplegia (one child), diplegia (five children), quadriplegia (two children), or dyskinesia (two children), and they were classified as Level I (three children) or II (seven children) on the Gross Motor Function Classification System (GMFCS) [38]. Children who had a significant illness, injury, or surgery within the previous six months that may have impacted their usual activity levels in the community, or was not possible to complete a three-dimentional gait analysis, were excluded from this study. Ethics approval was obtained from the local institutional review board and the University of Auckland Human Participants Ethics Committee. Written consent was obtained from each child’s parent or guardian along with assent from the child. All of the ten children were able to independently undergo gait analysis without any assistance from other people or walking aids.

### 2.2. Data Collection

**Figure 1:**
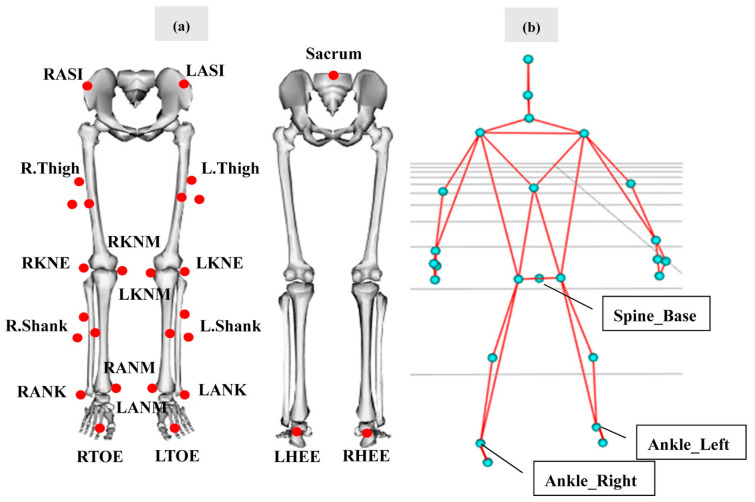
Lower limb reflective marker locations of a modified Cleveland Clinic marker set (**a**) and joint landmarks recorded by the Kinect V2 sensor (**b**).

As illustrated in Figure 1a, reflective markers were placed on the participants’ lower limb according to a modified Cleveland Clinic marker set [39,40]. This markerset consisted of the sacrum, right/left anterior superior iliac spine (RASI or LASI), rigid thigh clusters of markers (R.Thigh or L.Thigh), lateral and medial condyles of the knee (RKNE or RKNM or LKNE or LKNM), rigid shank clusters of markers (R.Shank/L.Shank), lateral and medial malleoli of the ankle (RANK or RANM or LANK or LANM), calcaneus (RHEE or LHEE), and the second metatarsal (RTOE or LTOE) of both feet.

An eight-infrared-camera motion capture system (Motion Analysis Corporation, Santa Rosa, CA, USA) recorded the reflective marker trajectories at a sampling rate of 100 Hz. The length of the calibrated motion capture volume was about 4 m. The Kinect V2 sensor (Microsoft Cop., Redmond, WA, USA) was placed on a tripod with a height of 0.8 m and a distance of 5 m from the start line of the walkway. The Kinect V2 sensor was placed in front of the participant to provide a frontal view, ensuring the tracking accuracy for both kinematic and spatiotemporal parameters. It was triggered simultaneously with the Motion Analysis system, recording coordinates of twenty-five skeletal landmarks (Figure 1b) at a fluctuating frequency around 30 Hz through a custom-written software program.

Three-dimensional gait analysis test consisted of two testing sessions. In the first testing session, participants were given sufficient time to try to walk along the walkway. After they became familiar with the testing procedures, they were asked to performed overground gait trials at a self-selected speed along the level walkway independently. Children walked from the starting line towards the Kinect V2 sensor barefoot. Each participant completed at least three successful gait trials. A successful gait trial should contain at least one complete right gait cycle with all the landmarks, and reflective markers could be seen in the motion capture interfaces of two systems. To test the interday reliability of the Kinect V2, participants were asked to attend another gait test session the following day. All the ten participants presented for the Kinect V2-based gait analysis in the second testing session. The experimental setup of the Kinect V2 for the second session was consistent with the previous one. At least three successful gait trials were obtained for each child. The criteria of a successful gait trial were consistent with what was given in the previous session.

### 2.3. Data Analysis

The data collected by the Kinect V2 and Motion Analysis system were filtered via a second-order Butterworth low-pass digital filter with a cut-off frequency of 6 Hz. Gait events were identified based on the distance between the sacrum and foot landmarks [41,42,43,44]. For the Motion Analysis system, the foot strike (FS) was identified as when the anterior-posterior (AP) distance between the heel of the leading foot and sacrum markers reached the maximum, and the toe-off (TO) was defined as when the AP distance between the second metatarsal marker of the rearfoot and the sacrum reached the maximum. For the Kinect V2, FS and TO events were detected similarly with the Motion Analysis, in which the “spine base” landmark was used as the sacrum, and the ankle landmark was used to represent the heel and second metatarsal due to its better tracking performance than the foot landmark. Spatiotemporal gait parameters were identified according to previous studies [35,45,46]. Definitions of selected spatiotemporal parameters in the Kinect V2 and Motion Analysis systems are presented in Table 1, and their calculation formulas are demonstrated in Table 2.

**Figure 2:**
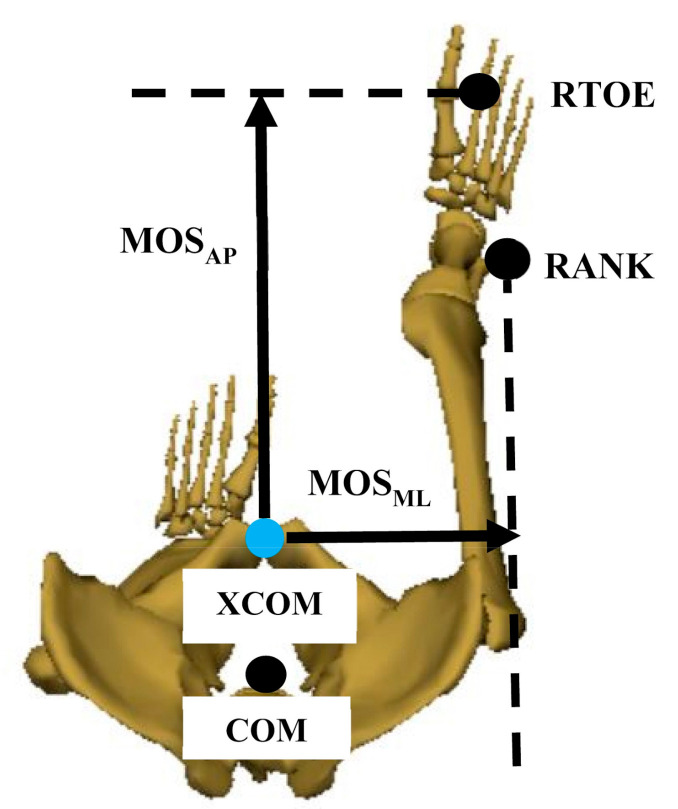
Illustration of the calculation of margin of stability (MOS).

The COM position was calculated as the average position among the sacrum, LASI, and RASI markers in the Motion Analysis [23,47]. The “spine_base” landmark position was considered as the COM in the Kinect V2 system (Figure 1b). The definition of MOS was presented in Figure 2.

Equations (7)–(9) were used to compute the dynamic MOS based on prior studies [21,23]:

(7) $$\omega_{0}\;=\;\sqrt{\frac{g}{l}}$$

where $\omega_{0}$ represented the eigenfrequency of the pendulum, $g\;=\;9.81\;m/s^{2}$ was the gravitational constant, and the l was the length of the pendulum, which was defined as the distance between COM and the heel marker (in Motion Analysis) or the ankle landmark (in the Kinect) at the foot strike.

(8) $$X_{COM}\;=\;a+\frac{v}{\omega_{0}}$$

where the $X_{COM}$ represented the extrapolated COM, a represented positions of COM in the AP or mediolateral (ML) direction. v was the velocity of COM, which was acquired by calculating the first time derivative of COM positions in the AP or ML direction.

(9) $$MOS\;=\;BOS-X_{COM}$$

MOS was finally obtained as the distance between XCOM and the boundary of BOS in the AP or the ML direction. BOS in the AP direction was identified using the second metatarsal marker in the AP direction of the leading foot, and BOS in the ML direction was estimated using the lateral malleoli marker in the ML direction of the leading foot [24]. MOS at foot strike, minimum MOS during the stance phase and MOS at midstance (the point when the swing limb passed the stance limb in the direction of progression) [48] were extracted for statistical analysis. All the gait event detections, spatiotemporal gait parameters, and MOS computations were conducted in customised scripts written in the Matlab R2019a (MathWorks Inc., Natick, MA, USA).

### 2.4. Statistics

In each gait trial, one right gait cycle was extracted for analysis. All the MOS and spatiotemporal parameters measured by each device on each testing day were averaged among three gait trials for each participant. The intraclass correlation coefficient (ICC) was used to assess the agreement between the Kinect V2 and Motion Analysis system and the agreement of the Kinect V2 between two testing days. ICCs were estimated, and their 95% confidential intervals were calculated based on a mean rating (k = 3), absolute-agreement, 2-way random-effects model. ICC values were interpreted as: excellent (0.75–1), modest (0.4–0.74), or poor (0–0.39) [49]. The standard error of measurement (SEM) was defined according to Equation (10):

(10) $$e\;=\;s\times \sqrt{1-r}$$

where e was SEM, s was the standard deviation of measurements determined from ANOVA [50,51], and r was ICC.

The relative error (in percentage) was computed to express the absolute between-device measurement difference as a percentage of the measure estimated by the referential Motion Analysis system (Equation (11)) [36].

(11) $$\eta \;=\;\frac{\left|\varepsilon_{Motion\;Analysis}-\varepsilon_{Kinect}\right|}{\varepsilon_{Motion\;Analysis}}\times 100\%$$

where η stood for the relative error (in percentage), $\varepsilon_{Motion\;Analysis}$ and $\varepsilon_{Kinect}$ were gait parameters measured by Motion Analysis and Kinect V2, respectively.

Furthermore, a Bland–Altman analysis of agreement [52] was performed between gait parameters obtained by the two devices. All the statistical analysis was conducted by using the SPSS statistical package version 25 (SPSS Inc., Chicago, IL, USA).

## 3. Results

**Figure 3:**
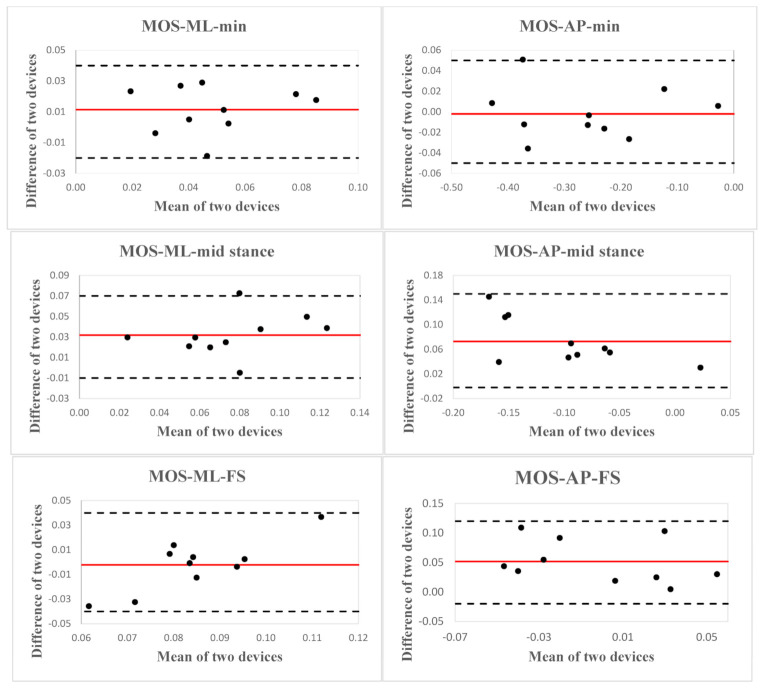
Bland–Altman plots indicate the agreement between MOS variables for ten children with cerebral palsy (CP) calculated using the Kinect V2 and Motion Analysis. The red solid line represents the reference line at the mean, and the two black dashed lines represent the upper and lower limit of agreement.

**Figure 4:**
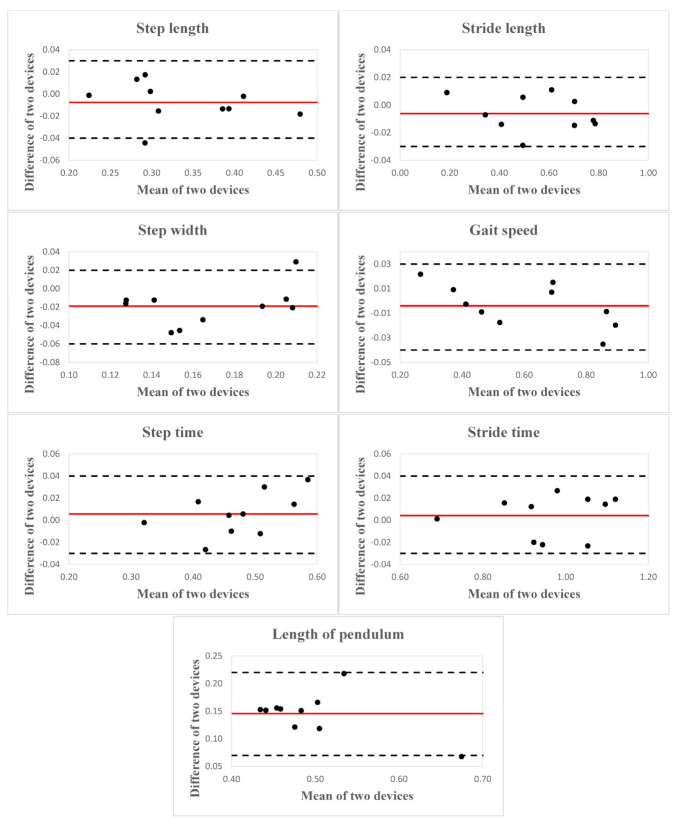
Bland–Altman plots indicate the agreement between gait spatiotemporal variables for ten children with CP calculated using the Kinect V2 and Motion Analysis. The red solid line represents the reference line at the mean, and the two black dashed lines represent the upper and lower limit of agreement.

Mean ± 1SD values for each spatial MOS and spatiotemporal gait parameters estimated by each device, inter-device ICC_2,k_ with 95% confidence intervals for agreement, SEM, and relative error (in percentage) are presented in Table 3. The Blan–Altman plots for every selected parameter are presented in Figure 3 and Figure 4; mean difference, LoA, upper and lower LoA are given in Table 4. Mean ± 1SD values for each spatial MOS and spatiotemporal gait parameters acquired by the Kinect V2 on two testing days, interday ICC_2,k_ with 95% confidence intervals for agreement and SEM are presented in Table 5.

For the MOS, an excellent agreement was observed for the minimum MOS during stance in both ML (ICC_2,k_ = 0.81) and AP (ICC_2,k_ = 0.99) directions, and a modest agreement (ICC_2,k_ = 0.42–0.68) was found for MOS at foot strike and midstance in both ML and AP directions between Motion Analysis and the Kinect V2. All the spatiotemporal gait parameters showed excellent agreement between the two devices (ICC_2,k_ = 0.83–0.99). A modest agreement was found in the equivalent pendulum length between the two devices (ICC_2,k_ = 0.45). Mean relative errors (in percentage) ranged from 1.75% to 186.4% for all the selected parameters. MOS at midstance and foot strike in the AP direction demonstrated the most deficient accuracy, with mean relative errors of 112.82% and 186.40%, respectively. All the selected spatiotemporal parameters exhibited lower relative errors (1.75–25.97%).

The interday reliability of most MOS variables was modest (ICC_2,k_ = 0.56–0.69) for the Kinect V2 except for the MOS at midstance in the ML direction, which showed low reliability between two test days (ICC_2,k_ = 0.28). All the spatiotemporal gait parameters (ICC_2,k_ = 0.78–0.88) and equivalent pendulum length (ICC_2,k_ = 0.92) showed excellent reliability between the two testing day.

## 4. Discussion

This study aimed to assess the validity and reliability of the Kinect V2 when using it to evaluate spatial MOS and spatiotemporal gait parameters during overground walking for children with CP. The results proved that the Kinect V2 could be employed as a valid and reliable screening tool to investigate spatiotemporal gait parameters and their progressive changes. For the MOS evaluation, the Kinect V2 could accurately assess the minimum MOS during the stance phase. These results partially supported our hypothesis. The Kinect V2′s overall moderate to poor interday reliability for MOS evaluation made it hard to distinguish the sources that caused the interday changes in MOS. Its reliability in MOS evaluation should be further verified before it can be widely adopted for in-clinic or home-based gait monitoring.

In this study, spatiotemporal variables derived from the Kinect V2 sensor showed an excellent agreement with their referential counterparts (ICC_2,k_ = 0.83–0.99). In accordance with the present results, previous studies have reported a reasonable validity of the Kinect V2 when assessing spatiotemporal gait parameters regardless of what the walking condition (overground or treadmill-based), walk speed (at a self-comfortable or specific speed), population (healthy or patients with movement disorders), and also the referential motion capture system (optoelectronic cameras, videos, or press sensing mat) was [35,36,44,53,54]. This study also demonstrated good reliability of the Kinect V2 sensor for spatiotemporal measurements (ICC_2,k_ = 0.78–0.88), favouring prior findings [35,55,56,57,58,59]. This study further enhanced the Kinect V2′s feasibility to be used as a valid and reliable tool to screen and investigate ongoing gait spatiotemporal progression for a pediatric population like children with CP.

However, except for the minimum MOS (ICC_2,k_ = 0.81–0.99), other MOS variables did not show such a strong agreement with those obtained by the referential motion capture system (ICC_2,k_ = 0.42–0.68). It was potentially attributed to two main reasons. Firstly, the length of the pendulum was calculated as the distance between COM and foot ground contact in a marker-based motion capture system [23]. In the Kinect V2-based gait analysis system, the ankle landmark was used to calculate MOS parameters instead of the foot centre due to its relatively stable tracking performance, which possibly resulted in a shorter pendulum length and affected the MOS estimation. Therefore, a moderate agreement was found between the length of pendulum calculated by the two motion capture devices (ICC_2,k_ = 0.45). Additionally, using the ankle landmark in MOS computation has the risk of underestimating BOS. A prior study reported that 66% of 492 patients with CP were affected by the intoeing abnormality, and around 25% of them had the out-toeing problem [60]. It indicates that the patients’ toes position medially or laterally with respect to the heel, leading to changes BOS boundaries. However, using the ankle landmark as an alternative to represent the BOS may fail to detect the foot progression during gait, leading to the interdevice deviations in MOS measurement.

Secondly, the MOS at foot strike and midstance were parameters that relied more heavily on the precise identification of related gait events. The Kinect was reported to show an error with ± 2 frames when detecting the heel strike and toe-off event for treadmill walking [41]. Its relatively low and fluctuating sampling frequency indicates that small deviations could lead to large gait detection errors. Two literature review studies reported that the Kinect’s automated body tracking algorithm was exceptionally good for some spatial gait parameters such as step length, width, and asymmetry when assessing the overground gait for young and old adult participants [32,33]. The difficulties in detecting gait events and relatively low and inconsistent sampling frequency hamper the precision of evaluating timing-related gait parameters, therefore giving the possibility of influencing the extraction of time-specific MOS parameters. Except for using the distance between the sacrum and ankle landmarks as the gait event detection criterion, previous studies have proposed various approaches to identify gait events, for example, utilising ankle or knee displacements that derived from depth images [54,61,62], extracting gait events based on some template models [63], identifying gait events according to the velocity of a specific landmark [64], and so on. Latorre et al. compared five gait event detection methods and found that the validity level varied among the selected methods for both healthy participants and stroke survivors when measuring spatiotemporal gait parameters [36]. Since MOS at midstance and mid-swing are significant indices for walking stability assessment [9,28], there is sufficient room for further progress in determining which gait event definition is the most appropriate one for a specific clinical group, such as children with CP, before the Kinect could be extrapolated to clinical applications.

Although the Kinect V2 could provide reliable interday spatiotemporal evaluation, it seems that the changes in clothing, accommodation to the testing procedure, and the patients’ performance over repeated gait trials may potentially result in the interday deviations in the MOS calculation (ICC_2,k_ = 0.28–0.69). De Jong et al. found that the test-retest ICCs of MOS are lower in healthy participants than patients with balance problems [65]. Since the ICC is determined by both between-subject variability and test-retest variability, a smaller between-subject variability in the healthy control group and similar test-retest variability may collectively result in lower ICCs [65]. In this study, the patient group was less homogeneous. Therefore, it could be induced that a comparable smaller interday variability may lead to lower ICCs in this study. Children in GMFCS Levels II and III exhibit lower within-session variability in their gait patterns when compared to children in GMFCS Level I [66]. Most of the participants of this study were classified in GMFCS Levels II, implying a higher variability of their gait between the two testing days. The MOS calculation seems to be sensitive to interday variabilities, making it hard to distinguish the sources resulting in changes in MOS. The Kinect V2 could provide accurate minimum MOS values (ICC_2,k_ = 0.81–0.99), which are essential gait metrics to assess the risk of falls [16]. However, its reliability of evaluating MOS should be further verified before utilising as a low-cost option to monitor fall risks during gait for children with CP.

From the perspective of clinical application, although the Kinect V2 is unable to provide precise and robust MOS estimation, it still has the potential to be utilised as a promising alternative tool for CP gait stability management. Firstly, the validity and reliability of Kinect V2-based gait spatiotemporal metrics are satisfactory. A previous systematic review study has investigated that some spatiotemporal parameters (i.e., double limb support time, step length, stride length, and step width) are useful features to distinguish CP gait stability from their typically developing peers [15]. Moreover, the gait speed is an essential index to evaluate the effectiveness of gait rehabilitation programmes for patients with CP [14,20]. Thus, the feasibility of the Kinect V2 to be applied as a cost-effective tool for assessing and monitoring gait stability for patients with CP has preliminarily warranted. Secondly, it is known that virtual reality (VR)-assisted gait training programmes enhance treatment outcomes by providing strong motivation and increasing concentration for participants [14]. Considering impaired sensory feedback networks associated with CP, multisensory feedback of an executed movement given by VR can furnish participants with enriching knowledge of performance [14,67]. Since the Kinect is advantageous in real-time motion sensing, it can be involved as an input device in VR-assisted gait stability training systems. Meanwhile, patients can learn and modify their postural control strategies with the help of the Kinect-based real-time gait stability assessment.

Except for the small sample size, some other limitations were encountered in this study. Firstly, to simplify the MOS calculation process, the “spine base” marker was used to represent the COM. However, the COM position with respect to the segmental coordinate system is generally predicted via regression equations with a full-body marker set in traditional optoelectronic motion capture systems [68]. It was found that COM positions decided by 7–9 multisegmental models allow a compromise between feasibility and accuracy when assessing the dynamic stability [69]. Similar anthropometric models and statistically equivalent serial chain methods have been developed to improve the COM estimation of the Kinect [70]. Further research should be undertaken to investigate the influence of different COM computation methods in COM displacement and MOS evaluation. Secondly, only one Kinect sensor was placed in front of the participants in this study to ensure the accuracy of both spatiotemporal and kinematic estimations. The single Kinect V2 setting has very limited capture volume, possibly resulting in missing data when falls occur outside the sensor’s field-of-view in a clinic or home-based environment. The application of multiple Kinect sensors presented a promising prospect to enlarge the volume for motion capture and solve occlusion problems [71]. Meanwhile, depth data obtained from the Kinect could be incorporated with other motion-sensing information, such as data recorded by accelerometers [72] and inertial sensors [73], to develop a multimodel gait stability assessment system for clinic or home-based monitoring. Thirdly, this study used ankle landmarks instead of foot landmarks to represent the BOS because the foot tracking is usually noisy and inaccurate in the Kinect V2. In contrast, the newest Azure Kinect sensor released in 2019 demonstrates significantly better foot tracking accuracy and more precise gait spatiotemporal parameter assessment, indicating improved image sensing strategies [74]. Therefore, in future investigations, it may be possible to use multiple Azure Kinect sensors to develop the precision of gait detection and skeleton tracking performance.

## 5. Conclusions

Results of this study reveal a reasonable validity and reliability level in evaluating spatiotemporal parameters and, in contrast, less strong validity and reliability when calculating MOS related variables, especially MOS at foot strike and midstance. The Kinect V2 was only firmly valid to provide minimum MOS measures, which is an essential gait metric to track the risk of falls [16]. These results comprehensively suggest that the Kinect V2 could be utilised as a low-cost, portable gait assessment tool to screen and observe ongoing gait progression for children with CP, especially for the spatiotemporal aspect. Future studies should involve more sensors and robust algorithms to enhance skeleton tracking, gait event detection performance, and MOS computation.

## Figures and Tables

**Table 1:** Definitions of spatiotemporal gait parameters in the Kinect V2 and Motion Analysis system [35,45,46].

	Motion Analysis	Kinect V2
Step Length (m)	Distance between the heel markers at the left and right foot strike	Distance between the ankles at the left and right foot strike
Stride Length (m)	Distance between RHEEs at the two consecutive right foot strike	Distance between the “ankle right” markers at the two consecutive right foot strike
Step Width (m)	Orthogonal distance from the LHEE to the vector formed by RHEEs in two consecutive foot strike	Orthogonal distance from the left ankles to the vector formed by the right ankles in two consecutive foot strike
Gait Speed (m/s)	Mean resultant velocity of the COM during the gait cycle	Mean resultant velocity of the “spine base” marker during the gait cycle
Step Time (s)	The time between the left and right foot strike	As per the Motion Analysis
Stride Time (s)	The time between two consecutive right foot strike	As per the Motion Analysis

**Table 2:** List of calculation formulas of spatiotemporal gait parameters in the Kinect V2 and Motion Analysis system [35,45,46]. 3D coordinates of RHEE in Motion Analysis or “right ankle” in Kinect V2 at the first and second right foot strike were represented by $\left(x_{1},y_{1,}z_{1}\right)$ and $\left(x_{3},y_{3,}z_{3}\right)$, respectively. 3D coordinates of LHEE in Motion Analysis or “left ankle” in Kinect V2 at the left foot strike were represented by $\left(x_{2},y_{2,}z_{2}\right)$. Average positions of the sacrum, LASI and RASI or “spine base” in the Kinect V2 were represented by $\left(x_{COM},y_{COM,}z_{COM}\right)$. $t_{1}$, $t_{2}$, and $t_{3}$ stood for time frames of first right foot strike, the left foot strike, and the second right foot strike. ρ stood for the sampling frequency of Motion Analysis or Kinect V2.

	Motion Analysis	
Step Length (m)	$\sqrt{{\left(x_{2}-x_{1}\right)}^{2}+{\left(y_{2}-y_{1}\right)}^{2}+{\left(z_{2}-z_{1}\right)}^{2}}$	(1)
Stride Length (m)	$\sqrt{{\left(x_{3}-x_{1}\right)}^{2}+{\left(y_{3}-y_{1}\right)}^{2}+{\left(z_{3}-z_{1}\right)}^{2}}$	(2)
Step Width (m)	$\frac{\left|\left(x_{2}-x_{1},y_{2}-y_{1},z_{2}-z_{1}\right)\times \left(x_{3}-x_{1},y_{3}-y_{1},z_{3}-z_{1}\right)\right|}{\left|\left(x_{3}-x_{1},y_{3}-y_{1},z_{3}-z_{1}\right)\right|}$	(3)
Gait Speed (m/s)	$\frac{\rho }{2(t_{3}-t_{1})}\overset{n}{\underset{i=1}{\sum }}\sqrt{{\left(x_{i+1,COM}-x_{i-1,COM}\right)}^{2}+{\left(y_{i+1,COM}-y_{i-1,COM}\right)}^{2}+{\left(z_{i+1,COM}-z_{i-1,COM}\right)}^{2}}$	(4)
Step Time (s)	$\frac{t_{2}-t_{1}}{\rho }$	(5)
Stride Time (s)	$\frac{t_{3}-t_{1}}{\rho }$	(6)

**Table 3:** Mean ± 1SD (n = 10) values for each MOS and spatiotemporal gait parameters collected by Motion Analysis and the Kinect on Day 1, intraclass correlation coefficient (ICC_2,k_) with 95% confidence intervals for agreement, SEM, and relative errors (in percentage) are provided. FS: foot strike; min: minimum; L: length of the pendulum.

	Motion Analysis	Kinect	ICC_2,k_	SEM	Relative Errors (%)
MOS-ML-min (m)	0.05 ± 0.02	0.04 ± 0.02	0.81 (0.20, 0.95)	0.004	32.04 ± 23.06
MOS-ML-mid stance (m)	0.09 ± 0.03	0.06 ± 0.03	0.68 (−0.25, 0.93)	0.01	37.06 ± 19.61
MOS-ML-FS (m)	0.08 ± 0.02	0.09 ± 0.01	0.42 (−1.78, 0.86)	0.004	22.06 ± 27.15
MOS-AP-min (m)	−0.26 ± 0.13	−0.26 ± 0.13	0.99 (0.96, 1.00)	0.03	9.83 ± 7.51
MOS-AP-mid stance (m)	−0.06 ± 0.05	−0.14 ± 0.07	0.66 (−0.21, 0.92)	0.06	112.82 ± 50.42
MOS-AP-FS (m)	0.02 ± 0.03	−0.03 ± 0.05	0.51 (−0.29, 0.86)	0.03	186.40 ± 163.49
Step Length (m)	0.33 ± 0.08	0.34 ± 0.08	0.99 (0.94, 0.99)	0.01	4.47 ± 4.62
Stride Length (m)	0.55 ± 0.20	0.55 ± 0.20	0.99 (0.99, 0.99)	0.02	2.49 ± 1.75
Step Width (m)	0.16 ± 0.04	0.18 ± 0.03	0.83 (0.17, 0.96)	0.01	16.81 ± 11.24
Gait Speed (m/s)	0.60 ± 0.22	0.60 ± 0.23	0.99 (0.99, 0.99)	0.03	2.69 ± 2.12
Step Time (s)	0.47 ± 0.08	0.47 ± 0.07	0.98 (0.94, 0.99)	0.01	3.23 ± 2.21
Stride Time (s)	0.97 ± 0.13	0.96 ± 0.13	0.99 (0.98, 0.99)	0.02	1.75 ± 0.72
L (m)	0.57 ± 0.06	0.42 ± 0.08	0.45 (−0.07, 0.85)	0.14	25.97 ± 6.84

**Table 4:** Results of Bland–Altman analysis of agreement between gait parameters calculated from the Kinect and Motion Analysis.

Parameter	Mean Difference	LoA	Lower LoA	Upper LoA
MOS-ML-min (m)	0.01	0.03	−0.02	0.04
MOS-ML-mid stance (m)	0.03	0.04	−0.01	0.07
MOS-ML-FS (m)	−0.002	0.04	−0.04	0.04
MOS-AP-min (m)	−0.002	0.05	−0.05	0.05
MOS-AP-mid stance (m)	0.07	0.08	−0.002	0.15
MOS-AP-FS (m)	0.05	0.07	−0.02	0.12
Step Length (m)	−0.01	0.03	−0.04	0.03
Stride Length (m)	−0.01	0.03	−0.03	0.02
Step Width (m)	−0.02	0.04	−0.06	0.02
Gait Speed (m/s)	−0.004	0.03	−0.04	0.03
Step Time (s)	0.01	0.04	−0.03	0.04
Stride Time (s)	0.004	0.04	−0.03	0.04
L (m)	0.15	0.08	0.07	0.22

**Table 5:** Mean ± 1SD (n = 10) values for each MOS and spatiotemporal gait parameters collected by the Kinect V2 on Day 1 and 2, ICC_2,k_ with 95% confidence intervals for agreement and SEM is provided. FS: foot strike; min: minimum; L: length of the pendulum.

	Day 1	Day 2	ICC_2,k_	SEM
MOS-ML-min (m)	0.03 ± 0.03	0.02 ± 0.03	0.63 (−0.22, 0.90)	0.01
MOS-ML-mid stance (m)	0.05 ± 0.04	0.03 ± 0.02	0.28 (−1.47, 0.81)	0.01
MOS-ML-FS (m)	0.08 ± 0.03	0.08 ± 0.03	0.56 (−1.01, 0.89)	0.01
MOS-AP-min (m)	−0.22 ± 0.11	−0.31 ± 0.10	0.64 (−0.24, 0.91)	0.15
MOS-AP-mid stance (m)	−0.17 ± 0.06	−0.14 ± 0.07	0.69 (−0.06, 0.92)	0.05
MOS-AP-FS (m)	−0.03 ± 0.07	0.01 ± 0.05	0.52 (−0.54, 0.87)	0.05
Step Length (m)	0.40 ± 0.08	0.40 ± 0.10	0.82 (0.23, 0.96)	0.06
Stride Length (m)	0.71 ± 0.19	0.70 ± 0.17	0.83 (0.27, 0.96)	0.24
Step Width (m)	0.17 ± 0.04	0.16 ± 0.04	0.88 (0.56, 0.97)	0.01
Gait Speed (m/s)	0.76 ± 0.23	0.76 ± 0.25	0.78 (0.06, 0.95)	0.49
Step Time (s)	0.49 ± 0.09	0.48 ± 0.06	0.85 (0.41, 0.96)	0.04
Stride Time (s)	1.00 ± 0.15	0.96 ± 0.13	0.82 (0.35, 0.96)	0.15
L (m)	0.44 ± 0.05	0.43 ± 0.04	0.92 (0.69, 0.98)	0.01

## Data Availability

The data presented in this study are available on request from the corresponding authors.
